# Treatment Outcomes of HIV Infected Children After Initiation of Antiretroviral Therapy in Southwest China: An Observational Cohort Study

**DOI:** 10.3389/fped.2022.916740

**Published:** 2022-07-12

**Authors:** Xiaoliang Zeng, Huanhuan Chen, Qiuying Zhu, Zhiyong Shen, Guanghua Lan, Jiangming Liang, Fuxiong Liang, Jinhui Zhu, Hui Xing, Yiming Shao, Yuhua Ruan, Jianfeng Zhang, Xiangjun Zhang

**Affiliations:** ^1^Guangxi Key Laboratory of Major Infectious Disease Prevention Control and Biosafety Emergency Response, Guangxi Center for Disease Control and Prevention, Nanning, China; ^2^The Second Affiliated Hospital of Guangxi Medical University, Nanning, China; ^3^State Key Laboratory of Infectious Disease Prevention and Control (SKLID), Collaborative Innovation Center for Diagnosis and Treatment of Infectious Diseases, Chinese Center for Disease Control and Prevention (China CDC), Beijing, China; ^4^Department of Public Health, University of Tennessee, Knoxville, TN, United States

**Keywords:** HIV, children, mortality, attrition, antiretroviral therapy

## Abstract

**Background:**

The number of HIV infected children receiving antiviral treatment in Guangxi is increasing. Understanding factors and trends of mortality and attrition in HIV-infected children under antiretroviral therapy (ART) was an urgent need to improve treatment outcomes. This study aimed to estimate mortality and attrition rates and identify factors that were associated with mortality and attrition after ART initiation among children with HIV in Guangxi, China between 2004 and 2018.

**Methods:**

Cohort study data were extracted from the National Free Antiretroviral Treatment Program (NFATP) database, which has standard guidelines for core treatment indicators and other data at all HIV/AIDS treatment facilities in Guangxi. A total of 901 HIV-infected children who have started ART were included in the study. The study collected the following data: age, gender, WHO clinic stages before ART, CD4 cell count before ART, Cotrimoxazole prophylaxis (CTX) use before ART, initial ART regimen, malnutrition before ART, abnormal liver function before ART, abnormal kidney function before ART, severe anemia before ART, and the time lag between an HIV diagnosis and ART initiation.

**Results:**

HIV-infected children under ART had a mortality rate of 0.87 per 100 person-years [95% Confidence Interval (CI) 0.63–1.11], and an attrition rate of 3.02 per 100 person-years (95% CI 2.57–3.47). Mortality was lower among children with a CD4 count between 200 and 500 copies/ml [Adjusted Hazard Ratio (AHR) 0.22, 95% CI 0.09–0.55], and CD4 count ≥500 copies/ml (AHR 0.10, 95% CI 0.03–0.29); but higher among children with late ART initiation at 1–3 months (AHR 2.30, 95% CI 1.07–4.94), and at ≥3 months (AHR 2.22, 95% CI 1.04–4.74). Attrition was lower among children with a CD4 count ≥500 copies/ml (AHR 0.62, 95% CI 0.41–0.95), but higher among children with late ART initiation at 1–3 months (AHR 1.55, 95% CI 1.05–2.30).

**Conclusion:**

Supportive programs are needed to educate children's families and parents on early ART, link HIV-infected children to care and retain them in care among other programs that treat and manage the medical conditions of HIV-infected children before ART initiation.

## Introduction

Despite intensive efforts for the prevention of mother-to-child transmission (MTCT), HIV consistently impacts children. Globally 1.8 million children aged <15 years living with HIV/AIDS, ~150,000 children became newly infected and 95,000 children died of HIV/AIDS-related illnesses in 2019 (1). Children with HIV disproportionally impacted middle- and low-income countries where the HIV disease burden was high and resources were limited (2, 3). China's MTCT prevention programs have achieved full coverage in 2015, although significant improvement has been made, the MTCT rate was only 5.7% in 2016 (4, 5). Moreover, the leading causes of death among infectious diseases in Chinese students aged 6–22 years shifted from rabies and tuberculosis in 2008 to HIV/AIDS in 2017 (6).

Without timely antiretroviral therapy (ART), the mortality of HIV-infected young children significantly increased (52.5 vs. 7.6% uninfected at the age of 2) (7). On the other hand, early HIV diagnosis and early ART initiation substantially reduced infant mortality (8). ART was recommended by the World Health Organization (WHO) for children infected with HIV regardless of age, CD4 status, and clinical stage as of 2015 (9). However, children's ART coverage remained low worldwide, only ~53% of children aged <15 years have received ART compared to 67% of people aged 15 and older in 2019 (10, 11). A nationwide study reported that as of 2018, only 5,892/8,029 (73.4%) HIV-infected children ≤14 years have started ART in China (12). Although ART had significantly extended children's survival time (12), HIV-infected children had much lower rates of the receipt of ART compared to the national average ART coverage of 80.4% in China (13). Furthermore, HIV-infected children might encounter challenges such as parental death, school dropout, and stigma which in turn resulted in negative consequences (7).

Southern and southwestern China reported the highest HIV disease burden nationwide (14). Guangxi Zhuang Autonomous Region which is located in southern China sharing borders with Thailand, Laos, and Myanmar (Golden Triangle) ranked 3^rd^ for HIV cases in 2018 (14, 15). It was one of six provinces and autonomous regions that had an HIV prevalence in pregnant women of over 50 per 100,000 population (14, 16). MTCT programs have been scaled up in Guangxi and contributed to a significant decrease in MTCT in recent years (17). Although early ART has been proven to be effective and beneficial for HIV-infected children, limited studies examined factors associated with death and attrition among HIV-infected children who were receiving ART. This retrospective study aimed to estimate mortality and attrition rates and identify factors that were related to death and attrition among a cohort of HIV-infected children who initiated ART. The findings of this study could be used to design programs with the goals to improve treatment outcomes and maximize the benefits of ART in HIV-infected children.

## Materials and Methods

### Study Design and Participants

The observational cohort study was conducted in Guangxi. Eligibility criteria included: (1) HIV positive children younger than 14 years old; (2) Enrolled in the National Free Antiretroviral Treatment Program (NFATP) between January 1, 2004, and December 31, 2018. All children or their parents or guardians were provided informed consent to the NFATP before they started the ART treatment. This NFATP informed consent also included encompassing their information in the NFATP database. NFATP included approximately all HIV-infected children who have been receiving ART in Guangxi. Furthermore, this study which only utilized de-identified data obtained approval from the institutional review board of the Guangxi Center for Disease Control and Prevention (GXIRB2016-0047-3). The study did not include participants who have not started ART.

### Data Collection

Cohort study data were extracted from the NFATP database. The following baseline data of study participants were collected: age, gender, clinic stage before ART (using WHO's criteria), CD4 count before ART, cotrimoxazole prophylaxis (CTX) use before ART, initial ART regimen, malnutrition status before ART, liver function before ART, kidney function before ART, anemia status before ART, and time between an HIV diagnosis and ART initiation. Follow-up data that were collected included: death, cessation of ART, and loss to follow-up. The date of death was based on death certificate information. Attrition was defined as lost to follow-up or cessation of ART as recorded in the NFATP database. Lost to follow-up was defined as missing more than 90 days after the date of the last ART clinic visit, which was also defined as the date of ART cessation. More details could be found in a previously published article that used the Chinese national HIV treatment cohort study databases (18).

China has been providing free ART for all HIV-infected individuals. The most common ART regimens that were prescribed to HIV-infected children were zidovudine (AZT), lamivudine (3TC), and nevirapine (NVP)/efavirenz (EFV)/lopinavir/ritonavir (LPV/r); or abacavir (ABC), lamivudine (3TC), and nevirapine (NVP)/efavirenz (EFV)/lopinavir/ritonavir (LPV/r). We categorized ART regimens into the following three groups: containing AZT, containing ABC, and others (19). Malnutrition was defined as BMI-for-age *Z*-score (BMIZ) <-3SD (standard deviation) which was recommended by WHO for children and adolescents (20). Abnormal liver function was defined as either alanine transaminase (ALT) or aspartate transaminase (AST) was higher than lab references. A cutoff point for AST and ALT level was set at 40 IU/L (21). Abnormal kidney function was defined when serum creatinine (Scr) was higher than the lab reference. Scr thresholds using the enzymatic method were set up for different age groups: 0–7 days, 1.19 mg/ml; 7 days−1 month, 0.79 mg/ml; 1 month−1 year, 0.50 mg/ml; 1–10 years, 1.09 mg/ml; and 10–19 years, 1.29 mg/ml (22). Severe anemia was defined as a <8 g/dl of hemoglobin concentration value (23).

### Statistical Analysis

We performed a time-to-event cohort analysis. The primary study endpoints were death and attrition. Mortality and attrition rates were calculated based on Poisson distributions and their 95% confidence intervals (CI) were assessed with incidence densities per 100 person-years at follow-up. Data were censored on Dec 31, 2019.

Cox proportional hazard models were performed to evaluate the treatment effect of initial ART regimens on death and attrition (cessation of ART or loss to follow-up) of HIV-infected children who started ART between 2004 and 2018, respectively. Competing risks for cause-specific hazard models were censored accordingly. The hazard ratios (HR) were generated through univariate regression models. Multivariate regression models were used to generate adjusted hazard ratios (AHR) with mortality and attrition, respectively. The following variables were included in the adjusted models: age before ART, gender, WHO stage before ART, CD4 count before ART, CTX use before ART, initial ART regimen, malnutrition before ART, abnormal liver function before ART, abnormal kidney function before ART, severe anemia before ART, time lag since HIV diagnosis and the start of ART, and year of ART initiation. A two-sided p-value of ≤0.05 was regarded as statistically significant. Statistical Analysis System (SAS 9.1™ for Windows; SAS Institute Inc., NC, USA) was used for all data analyses.

## Results

### Baseline Characteristics of Study Participants

Of 911HIV-infected children who started ART from 2004 to 2018 in Guangxi, 10 participants were older than 14 years at ART initiation. A total of 901 study participants were included in the final cohort study analyses. The sample's mean age was 5.1 years with an SD of 3.2. Here was the age distribution of participants who were not older than 5 years, ≤1, 13.3% (67); 1–2, 20.9% (105); 2–3, 23.1% (116); 3–4, 21.4% (108); and 4–5, 21.3% (107). Regarding participants' characteristics at ART initiation, 55.8% of participants were <5 years old, more than half of participants were males (52.3%), and more than one-third were at a clinic stage of III or IV (35.1%, Table 1). In general, participants showed a low CD4 count level before ART initiation, many of them used CTX before ART initiation (52.2%), and the majority used ART regimens that contained AZT (71.6%). Furthermore, many HIV-infected children had medical conditions of malnutrition, abnormal liver function, abnormal kidney function, and severe anemia (6.0, 43.6, 14.9, and 4.1%, respectively). More than one-third of participants initiated ART within 1 month after receiving an HIV diagnosis (36.5%).

**Table 1:** Characteristics of HIV infected children started ART in Guangxi, 2004–2018.

**Variable**	**Number**	**%**
Total	901	100.0
**Age before ART**		
0–5 years	503	55.8
≥5 years	398	44.2
**Gender**		
Male	471	52.3
Female	430	47.7
**WHO stage before ART**		
1/2	585	64.9
$\frac{3}{4}$	316	35.1
**CD4 count before ART**		
<200 copies/ml	342	38.0
200–500 copies/ml	225	25.0
≥500 copies/ml	334	37.0
**CTX use before ART**		
No	431	47.8
Yes	470	52.2
**Initial ART regimen**		
Containing AZT	645	71.6
Containing ABC	185	20.5
Others	71	7.9
**Malnutrition before ART**		
No	847	94.0
Yes	54	6.0
**Abnormal liver function before ART**		
No	508	56.4
Yes	393	43.6
**Abnormal kidney function before ART**		
No	767	85.1
Yes	134	14.9
**Severe anemia before ART**		
No	864	95.9
Yes	37	4.1
**Time lag since HIV diagnosis and start of ART**		
<1 month	329	36.5
1–3 months	244	27.1
≥3 months	328	36.4
**Year of ART initiation**		
2004–2008	177	19.6
2009–2011	217	24.1
2012–2018	507	56.3

### Mortality

**Figure 1:**
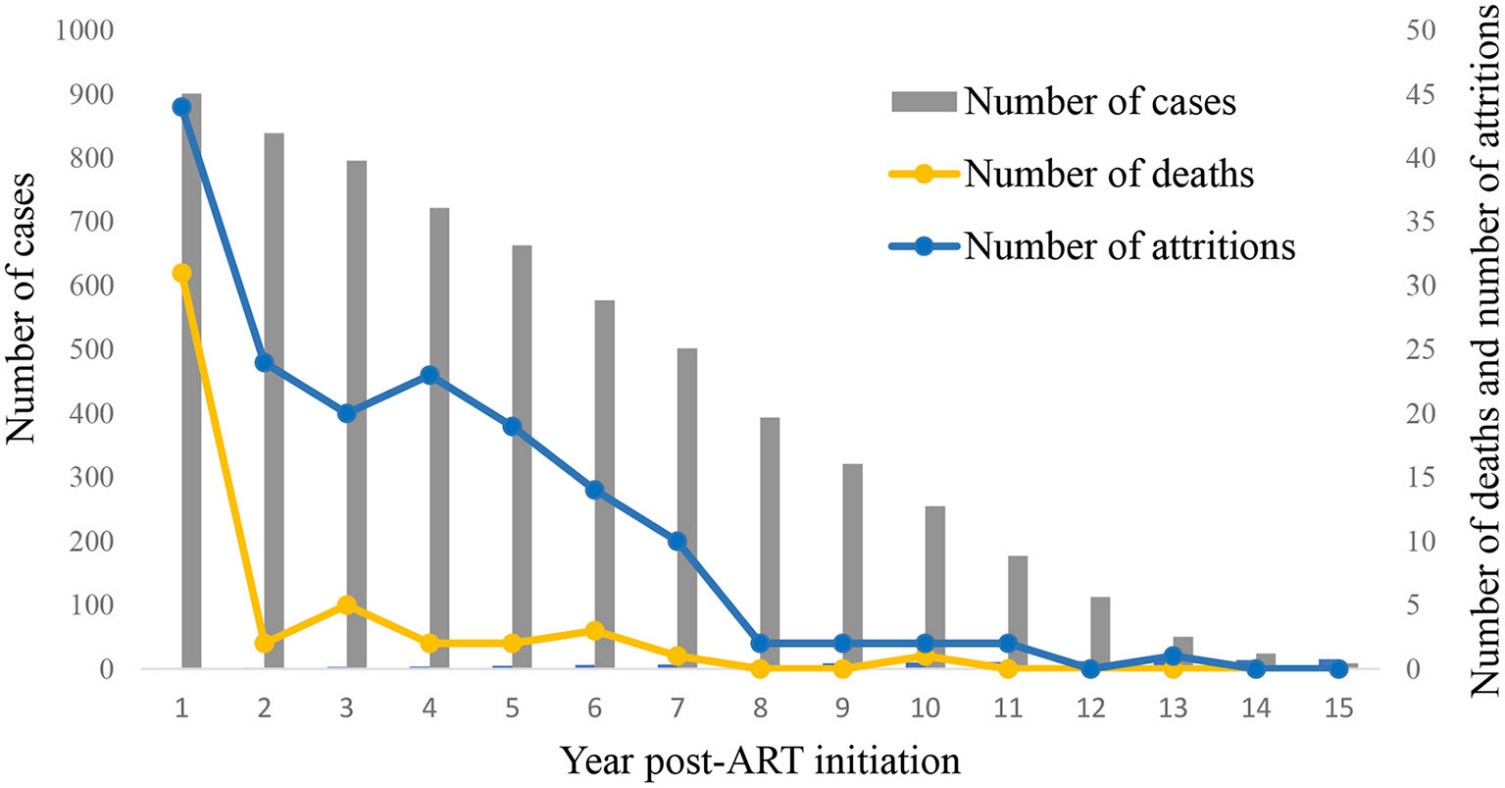
Mortality and attrition rates of HIV-infected children started ART in Guangxi, 2004–2018.

Among 901 HIV-infected children who started ART between 2004 and 2018, 47 of them died, the average mortality rate was 0.87 deaths per 100 person-years (95% CI 0.63–1.11). The mortality rate per 100 person-years was 3.65 in the first year of ART (95% CI 2.40–4.90), and it decreased thereafter (Figure 1 and Supplementary Table). Univariate Cox regression analyses indicated that several factors were significantly associated with mortality, including status before ART started such as disease severity (WHO clinic stage), CD4 count, malnutrition, abnormal liver function, initial ART regimen, and the time lag between an HIV diagnosis and ART initiation. In the multivariate model, higher levels of CD4 count before ART initiation were associated with reduced risks of mortality (200–500, AHR 0.22, 95% CI 0.09–0.55; ≥500, AHR 0.10, 95% CI 0.03–0.29, Table 2). Furthermore, a longer time lag between an HIV diagnosis and ART initiation was associated with increased risks of mortality (1–3 months, AHR 2.30, 95% CI 1.07–4.94; ≥3 months, AHR 2.22, 95% CI 1.04–4.74).

**Table 2:** Mortality rates and risk factors of HIV infected children started ART in Guangxi, 2004–2018.

**Variable**	**Number**	**Deaths**	**Person-years**	**Deaths per 100 person-years (95% CI)**	**HR (95%CI)**	***p*-Value**	**AHR (95%CI)**	***p*-Value**
Total	901	47	5,394.48	0.87 (0.63–1.11)				
**Age before ART**								
0–5 years	503	25	3,525.65	0.71 (0.44–0.98)	Reference		Reference	
≥5 years	398	22	1,868.83	1.18 (0.70–1.66)	1.24 (0.69–2.20)	0.473	0.71 (0.38–1.34)	0.293
**Gender**								
Male	471	21	2,948.97	0.71 (0.42–1.01)	Reference		Reference	
Female	430	26	2,445.51	1.06 (0.66–1.46)	1.39 (0.78–2.46)	0.267	1.77 (0.97–3.23)	0.064
**WHO stage before ART**								
1/2	585	20	3,457.10	0.58 (0.33–0.83)	Reference		Reference	
3/4	316	27	1,937.39	1.39 (0.88–1.91)	2.56 (1.44–4.57)	0.001	1.44 (0.75–2.75)	0.269
**CD4 count before ART**								
<200 copies/ml	342	37	1,857.02	1.99 (1.37–2.62)	Reference		Reference	
200–500 copies/ml	225	6	1,430.9	0.42 (0.09–0.75)	0.23 (0.1–0.54)	0.001	0.22 (0.09–0.55)	0.001
≥500 copies/ml	334	4	2,106.56	0.19 (0.01–0.37)	0.10 (0.04–0.29)	<0.001	0.10 (0.03–0.29)	<0.001
**CTX use before ART**								
No	431	19	2,107.14	0.90 (0.51–1.30)	Reference		Reference	
Yes	470	28	3,287.34	0.85 (0.54–1.16)	1.23 (0.68–2.20)	0.495	0.60 (0.31–1.17)	0.132
**Initial ART regimen**								
Containing AZT	645	27	4,104.26	0.66 (0.42–0.90)	Reference		Reference	
Containing ABC	185	12	836.28	1.43 (0.64–2.23)	1.69 (0.85–3.34)	0.132	1.17 (0.54–2.55)	0.693
Others	71	8	453.94	1.76 (0.57–2.95)	2.77 (1.26–6.09)	0.012	1.49 (0.62–3.57)	0.374
**Malnutrition before ART**								
No	847	41	5,104.40	0.80 (0.56–1.04)	Reference		Reference	
Yes	54	6	290.08	2.07 (0.45–3.68)	2.45 (1.04–5.76)	0.041	1.84 (0.74–4.56)	0.188
**Abnormal liver function before ART**								
No	508	20	2,826.21	0.71 (0.41–1.01)	Reference		Reference	
Yes	393	27	2,568.27	1.05 (0.66–1.44)	1.71 (0.96–3.05)	0.07	1.36 (0.73–2.51)	0.331
**Abnormal kidney function before ART**								
No	767	37	4,316.97	0.86 (0.59–1.13)	Reference		Reference	
Yes	134	10	1,077.51	0.93 (0.37–1.49)	1.41 (0.70–2.86)	0.334	1.23 (0.54–2.80)	0.615
**Severe anemia before ART**								
No	864	44	5,122.72	0.86 (0.61–1.11)	Reference		Reference	
Yes	37	3	271.76	1.10 (0.00–2.32)	1.47 (0.46–4.75)	0.516	1.46 (0.41–5.16)	0.557
**Time lag since HIV diagnosis and the start of ART**								
<1 month	329	11	2,091.88	0.53 (0.22–0.83)	Reference		Reference	
1–3 months	244	17	1,433.91	1.19 (0.64–1.74)	2.14 (1.00–4.58)	0.049	2.30 (1.07–4.94)	0.033
≥3 months	328	19	1,868.69	1.02 (0.57–1.46)	1.77 (0.84–3.71)	0.134	2.22 (1.04–4.74)	0.04
**Year of ART initiation**								
2004–2008	177	10	1,707.5	0.59 (0.23–0.94)	Reference		Reference	
2009–2011	217	14	1,579.67	0.89 (0.43–1.34)	1.24 (0.55–2.8)	0.606	1.22 (0.50–2.95)	0.667
2012–2018	507	23	2,107.31	1.09 (0.66–1.53)	1.01 (0.47–2.14)	0.989	1.18 (0.45–3.12)	0.736

*HR, hazard ratio in univariate regression; AHR, adjusted hazard ratio in multivariate regression, the adjusted model included all 12 variables in this table*.

### Attrition

Among 901 HIV-infected children who started ART between 2004 and 2018, 163 attritions were reported, and the average attrition rate was 3.02 per 100 person-years (95% CI 2.57–3.47). The attrition rate per 100 person-years was 5.18 in the first year of ART (95% CI 3.69–6.67) and decreased after then (Figure 1 and Supplementary Table). We identified the following factors that were significantly associated with attrition using the univariate Cox regression model: age before ART, disease severity (WHO clinic stage) before ART, CD4 count before ART, initial ART regimen, and the time lag between an HIV diagnosis and ART initiation. In the multivariate model, a high CD4 level before ART initiation was associated with a reduced risk of attrition rate (≥500, AHR 0.62; CI 0.41–0.95, Table 3). On the other hand, delayed initiation of ART (1–3 months) was associated with an increased risk of attrition (AHR 1.55, CI 1.05–2.30).

**Table 3:** Attrition rates and risk factors of HIV infected children started ART in Guangxi, 2004–2018.

**Variable**	**Number**	**Attritions**	**Person-years**	**Attritions per 100 person-years (95% CI)**	**HR (95%CI)**	***p*-Value**	**AHR (95%CI)**	***p*-Value**
Total	901	163	5,394.48	3.02 (2.57–3.47)				
**Age before ART**								
0–5 years	503	84	3,525.65	2.38 (1.89–2.88)	Reference		Reference	
≥5 years	398	79	1,868.83	4.23 (3.32–5.14)	1.56 (1.14–2.13)	0.005	1.32 (0.92–1.89)	0.131
**Gender**								
Male	471	76	2,948.97	2.58 (2.01–3.14)	Reference		Reference	
Female	430	87	2,445.51	3.56 (2.83–4.29)	1.33 (0.98–1.81)	0.069	1.39 (1.01–1.91)	0.041
**WHO stage before ART**								
1/2	585	94	3,457.1	2.72 (2.18–3.25)	Reference		Reference	
3/4	316	69	1,937.39	3.56 (2.74–4.38)	1.37 (1.01–1.88)	0.046	1.22 (0.86–1.73)	0.277
**CD4 count before ART**								
<200 copies/ml	342	77	1,857.02	4.15 (3.24–5.05)	Reference		Reference	
200–500 copies/ml	225	38	1,430.9	2.66 (1.83–3.48)	0.65 (0.44–0.96)	0.030	0.66 (0.44–1.01)	0.053
≥500 copies/ml	334	48	2,106.56	2.28 (1.65–2.91)	0.56 (0.39–0.81)	0.002	0.62 (0.41–0.95)	0.026
**CTX use before ART**								
No	431	70	2,107.14	3.32 (2.56–4.08)	Reference		Reference	
Yes	470	93	3,287.34	2.83 (2.27–3.39)	0.96 (0.70–1.31)	0.793	0.87 (0.60–1.24)	0.430
**Initial ART regimen**								
Containing AZT	645	115	4,104.26	2.80 (2.30–3.30)	Reference		Reference	
Containing ABC	185	24	836.28	2.87 (1.75–3.99)	0.89 (0.57–1.38)	0.602	0.75 (0.47–1.20)	0.231
Others	71	24	453.94	5.29 (3.22–7.35)	1.97 (1.27–3.06)	0.003	1.72 (1.06–2.78)	0.027
**Malnutrition before ART**								
No	847	155	5,104.40	3.04 (2.57–3.50)	Reference		Reference	
Yes	54	8	290.08	2.76 (0.89–4.62)	0.92 (0.45–1.86)	0.808	0.79 (0.38–1.64)	0.53
**Abnormal liver function before ART**								
No	508	85	2,826.21	3.01 (2.38–3.63)	Reference		Reference	
Yes	393	78	2,568.27	3.04 (2.38–3.69)	1.08 (0.79–1.47)	0.619	1.07 (0.77–1.49)	0.686
**Abnormal kidney function before ART**								
No	767	134	4,316.97	3.10 (2.59–3.62)	Reference		Reference	
Yes	134	29	1,077.51	2.69 (1.74–3.65)	0.99 (0.66–1.49)	0.968	1.00 (0.63–1.59)	0.996
**Severe anemia before ART**								
No	864	159	5,122.72	3.10 (2.63–3.57)	Reference		Reference	
Yes	37	4	271.76	1.47 (0.07–2.88)	0.50 (0.19–1.36)	0.176	0.53 (0.19–1.48)	0.226
**Time lag since HIV diagnosis and the start of ART**								
<1 month	329	49	2,091.88	2.34 (1.70–2.98)	Reference		Reference	
1–3 months	244	53	1,433.91	3.70 (2.73–4.67)	1.54 (1.04–2.27)	0.030	1.55 (1.05–2.30)	0.029
≥3 months	328	61	1,868.69	3.26 (2.47–4.06)	1.30 (0.89–1.90)	0.169	1.19 (0.81–1.77)	0.378
**Year of ART initiation**								
2004–2008	177	41	1,707.5	2.4 (1.68–3.12)	Reference		Reference	
2009–2011	217	46	1,579.67	2.91 (2.09–3.73)	1.11 (0.72–1.71)	0.627	1.13 (0.70–1.80)	0.622
2012–2018	507	76	2,107.31	3.61 (2.82–4.4)	1.12 (0.75–1.67)	0.588	1.19 (0.71–1.98)	0.505

*HR, hazard ratio in univariate regression; AHR, adjusted hazard ratio in multivariate regression, the adjusted model included all 12 variables in this table*.

## Discussion

The study reported a mortality rate of 0.87 per 100 person-years in Guangxi HIV-infected children who have started ART. This result was lower than comparable reports from other Asian regions (2.1–2.86 per 100 person-years) for HIV-infected children (24, 25). Also, this mortality rate was much lower than in HIV-infected adults who were receiving ART in Guangxi (5.94 per 100 person-years) (26, 27). Moreover, mortality reached the peak of 3.65 per 100 person-years within the first year, dramatically reduced from the 2^nd^ year, and reached nearly 0 at the 8th year of ART initiation. The trends were consistent with previous studies that reported generally higher mortalities in the first 3–6 months of ART initiation and decreased over time (24, 28). It could be explained that infections and other complications related to advanced HIV disease and late clinic stages were the possible major causes of the first-year deaths because medication-related deaths were generally low (28).

ART has significantly reduced HIV-infected children's mortality in this sample and initiating ART at a high CD4 level of ≥500 contributed to the lowest mortality. On the other hand, low CD4 count before ART initiation and late ART initiation resulted in increased mortality. In addition to a weakened immune system and its consequences, previous studies reported that a higher CD4 count before ART initiation could reduce the odds of virologic failure and HIV drug resistance mutations (29). However, 63.5% of participants have started ART after 1 month of an HIV diagnosis, and 38% started ART with a very low CD4 count (<200 copies/ml) in this sample. And at the time of initiating ART, many children were with other medical conditions, such as malnutrition, abnormal liver function, abnormal kidney function, and severe anemia. It suggested that supportive programs, such as nutritional support, routine examination, and lab test are needed in addition to ART promotion.

The overall attrition rate was 3.02 per 100 person-years in this study (18.1%). The result was consistent with the reports of Thailand (2.9 per 100 person-years) and Zambia (16%) for HIV-infected children (25, 30), but much lower than HIV-infected adults who started ART in Guangxi (10.86 per 100 person-years) (26). More than 25% of attritions occurred in the first year of ART initiation. High attrition rates can result in suboptimal treatment outcomes, increased mortality, increased cost of care, and excess infections (31). Therefore, future interventions can provide retention support, such as case management, and follow-ups to help retain HIV-infected children in care. Moreover, health education and linkage to care services are needed to educate families with HIV-infected children to start ART as early as possible and remove possible barriers for ART initiation.

One strength of the study was it utilized a database that included approximately all HIV-infected children who were under ART in Guangxi. The study provided a profile of this group of children and assessed factors associated with mortality and attrition. The study also confirmed the effectiveness of pediatric ART. Therefore, the findings have important real-world implications to reduce mortality and attrition in HIV-infected children. The study has limitations. First, the study included HIV-infected children who were in NFATP between 2004 and 2018 in Guangxi, some groups were not covered such as HIV-infected children who were not on ART. Therefore, the results cannot be generalized to these groups. Second, this sample and the number of events were relatively small (47 deaths), which possibly could cause the overfitting of the Cox model to predict risk factors for mortality. Third, the retrospective study was limited by the existing information in the database. Some information that might be helpful to explain the study results was lacking, such as children's viral load, parental HIV status, and parental or families' attitudes or sociodemographic characteristics. It is possible the lack of these variables results in unexpected results such as the higher mortality/attrition rates for the 1–3 months of ART initiation group than the >3 months group.

## Conclusion

MTCT prevention efforts and ART have significantly reduced HIV-infected children's mortality although first-year mortality and attrition were relatively high after ART initiation. Low CD4 count and late ART initiation (>1 month) were associated with increased mortality and attrition. The findings have implications to design interventions and programs that support linkage to care, early ART initiation, and retention in care.

## Data Availability Statement

The data analyzed in this study is subject to the following licenses/restrictions. Data were extracted from the National Free Antiretroviral Treatment Program database. The original database was not available to the public due to institutional regulations. Requests to access these datasets should be directed to JZha, zhangjianfeng@stu.gxmu.edu.cn.

## Ethics Statement

This study was approved by the Institutional Review Board of the Guangxi Center for Disease Control and Prevention (GXIRB2016-0047-3). Written informed consent from the participants' legal guardian/next of kin was not required to participate in this study in accordance with the national legislation and the institutional requirements.

## Author Contributions

XZe, JZha, and YR were responsible for study design and planning. QZ, ZS, GL, and HC contributed to the data collection and management. XZh, JZhu, and YR contributed to the data analyses. XZe, JZha, YS, XZh, and YR contributed to the interpretation of the study results. XZe, JZha, XZh, and YR contributed to writing the report. All authors reviewed and revised the manuscript and approved the final version of the manuscript.

## Funding

This work was supported by National Natural Science Foundation of China (82160636 and 11971479), Guangxi Natural Science Foundation Project (grants 2020GXNSFAA159020), Guangxi Key Laboratory of AIDS Prevention Control and Translation (ZZH2020010), Ministry of Science and Technology of China (2018ZX10721102-006 and 2018ZX10715008), Guangxi Bagui Honor Scholarship, and Chinese State Key Laboratory of Infectious Disease Prevention and Control. The funders of the study had no role in the study design, data collection, data analysis, data interpretation, and writing of the paper. The corresponding author (JZha) has full access to all data in the study and takes final responsibility for the decision to submit it for publication.

## Conflict of Interest

The authors declare that the research was conducted in the absence of any commercial or financial relationships that could be construed as a potential conflict of interest.

## Publisher's Note

All claims expressed in this article are solely those of the authors and do not necessarily represent those of their affiliated organizations, or those of the publisher, the editors and the reviewers. Any product that may be evaluated in this article, or claim that may be made by its manufacturer, is not guaranteed or endorsed by the publisher.

## Abbreviations

AHR: adjusted hazard ratio
HR: hazard ratio.
