# Supplementary material for: Treatment Outcomes of HIV Infected Children After Initiation of Antiretroviral Therapy in Southwest China: An Observational Cohort Study
Source: Front Pediatr. 2022 Jul 12;10:916740. doi: 10.3389/fped.2022.916740 (PMC9315248; doi:10.3389/fped.2022.916740)
Supplement: Supplementary file 1 [file Table_1.DOCX]

Supplementary Table.

Mortality and attrition rates of HIV infected children started ART in Guangxi, 2004-2018

| Variable | Number | Deaths | Person years | Deaths per 100 person years (95% CI) | Attritions | Attritions per 100 person years (95% CI) |
| --- | --- | --- | --- | --- | --- | --- |
| Total | 901 | 47 | 5394.48 | 0.87(0.63-1.11) | 163 | 3.02(2.57-3.47) |
| Year post-ART initiation |  |  |  |  |  |  |
| The first year | 901 | 31 | 849.66 | 3.65(2.40-4.90) | 44 | 5.18(3.69-6.67) |
| The second year | 839 | 2 | 825.08 | 0.24(0.00-0.57) | 24 | 2.91(1.77-4.04) |
| The third year | 796 | 5 | 780.41 | 0.64(0.09-1.19) | 20 | 2.56(1.47-3.66) |
| The fourth year | 722 | 2 | 705.41 | 0.28(0.00-0.67) | 23 | 3.26(1.96-4.56) |
| The 5th year | 663 | 2 | 652.58 | 0.31(0.00-0.72) | 19 | 2.91(1.64-4.19) |
| The 6th year | 577 | 3 | 566.22 | 0.53(0.00-1.11) | 14 | 2.47(1.21-3.74) |
| The 7th year | 502 | 1 | 488.53 | 0.20(0.00-0.60) | 10 | 2.05(0.81-3.28) |
| The 8th year | 394 | 0 | 389.23 | - | 2 | 0.51(0.00-1.21) |
| The 9th year | 321 | 0 | 316.96 | - | 2 | 0.63(0.00-1.48) |
| The 10th year | 255 | 1 | 247.42 | 0.40(0.00-1.18) | 2 | 0.81(0.00-1.90) |
| The 11th year | 177 | 0 | 167.39 | - | 2 | 1.19(0.00-2.81) |
| The 12th year | 113 | 0 | 109.48 | - | 0 | - |
| The 13th year | 50 | 0 | 47.32 | - | 1 | 2.11(0.00-6.15) |
| The 14th year | 24 | 0 | 22.88 | - | 0 | - |
| The 15th year | 9 | 0 | 8.55 | - | 0 | - |
